# Effects of lower versus higher oxygen targets on out-of-hospital cardiac arrest

**DOI:** 10.1186/s13054-023-04740-y

**Published:** 2023-12-01

**Authors:** Yang Zhao, Qian Wang, Bin Zang

**Affiliations:** 1 Department of Critical Care Medicine, Shengjing Hospital of China Medical University, Shenyang, 110000 China; 2 Department of Emergency, The Fourth Affiliated Hospital of China Medical University, Shenyang, 110000 China

To the Editor,

We have read with interest the published article entitled "Lower versus higher oxygen targets for out-of-hospital cardiac arrest: a systematic review and meta-analysis" by Cheng et al. [1]. The article is well written, and I have two concerns as explained below.

First, there may be a need for some data to be revised. The numbers in Table 1 [1] for sample size in the study by Bernard et al. [2] differ from those in the forest plot of Fig. 2 [1] (425 vs. 401). In the original text, Bernard et al. mentioned that hospital mortality should be 132 deaths in the lower oxygen saturation targets group (*n* = 214) and 109 deaths in the higher oxygen saturation targets group (*n* = 211), which is different from what the authors mentioned in the article. After correcting this error, the combined mortality rate should be RR, 0.97; 95% CI 0.80–1.17; *p* = 0.72 (Fig. 1).

**Fig. 1:**
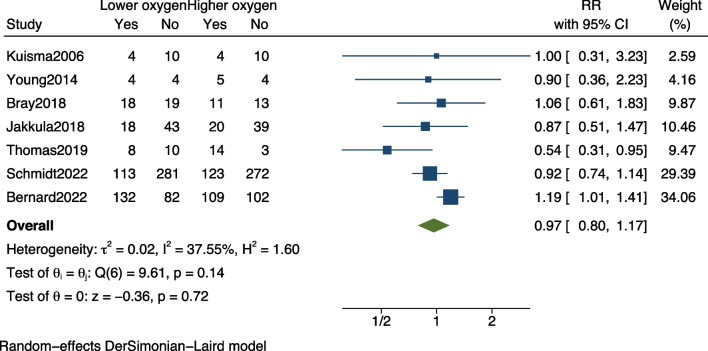
Forest plot of pooled mortality. Lower oxygen target versus higher oxygen target among out-of-hospital cardiac arrest patients. The points and the bars represent the relative risk (RR) and 95% confidence interval (CI). RR, relative risk; CI, confidence interval

Second, this study used 90-day mortality as its primary outcome. In cases where data for 90-day mortality were unavailable, the study also considered mortality at 30 days or in-hospital mortality for the pooled analysis. It is worth noting that among the seven included randomized controlled trials (RCTs), only two studies reported 90-day mortality [3, 4], one reported 30-day mortality [5], and the remaining four studies reported hospital mortality [2, 6–8]. Combining these different time points for mortality outcomes may increase the overall sample size and statistical power. Nevertheless, it could also introduce heterogeneity, affect the interpretation of the findings, and impact the reliability of the conclusion drawn.

I reanalyzed the data, excluding three studies, and performed a new analysis on the remaining four studies with the endpoint of hospital mortality [2, 6–8]. The results indicate that a higher oxygen target significantly reduces hospital mortality among out-of-hospital cardiac arrest patients (RR, 1.17; 95% CI 1.00–1.37; *p* = 0.05) (Fig. 2). Although the results did not demonstrate significant heterogeneity, the substantial weight of the Bernard study suggests that further studies are urged to offer more definitive answers.

**Fig. 2:**
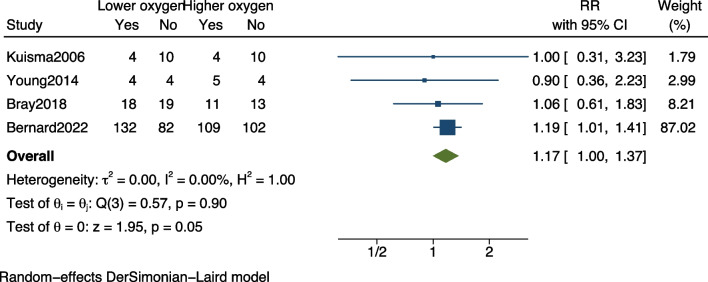
Forest plot of hospital mortality. Lower oxygen target versus higher oxygen target among out-of-hospital cardiac arrest patients. The points and the bars represent the relative risk (RR) and 95% confidence interval (CI). RR, relative risk; CI, confidence interval

## Data Availability

Not applicable.
